# Gyroscopic Radiosurgery in a Case of Juxtapapillary Choroidal Melanoma: A Case Report and Review of the Literature

**DOI:** 10.7759/cureus.88776

**Published:** 2025-07-25

**Authors:** Rohit K Chaini, Sudheer K Tyagi, Soumya Subhadarsini, Sapna V Manocha, Ganesh K Jadhav, Sunil Chauhan, Harish Kumar

**Affiliations:** 1 Radiation Oncology, Indraprastha Apollo Hospital, New Delhi, IND; 2 Neurological Surgery, Indraprastha Apollo Hospital, New Delhi, IND

**Keywords:** choroidal melanoma, grs, gyroscopic radiosurgery, juxtapapillary, ocular melanoma, srs, stereotactic radiosurgery, zap-x

## Abstract

The management of localized choroidal melanoma generally involves definitive radiation therapy when globe preservation is feasible. Stereotactic radiosurgery (SRS) using the Gamma Knife system (Elekta AB, Stockholm, Sweden) or the CyberKnife system (Accuray Inc., Madison, WI, USA) is a well-established radiation modality for treating choroidal melanoma, with outcomes comparable to those of other radiation techniques. The ZAP-X Gyroscopic Radiosurgery system (ZAP Surgical Systems Inc., San Carlos, CA, USA) is a more recent platform designed for cranial SRS treatments. It may be considered a substitute for existing SRS modalities due to a similar dosimetric profile. Here, we report a case of choroidal melanoma treated using ZAP-X gyroscopic radiosurgery (GRS).

A 46-year-old female presented with a medium-sized juxtapapillary choroidal melanoma in the left eye. The tumor was located nasal to the optic disc and involved the nasal half of the disc. On B-scan ultrasonography, the lesion measured 9.27 mm in its largest basal diameter and 5.55 mm in thickness. The gross tumor volume (GTV) was calculated at 0.391 cm³. A 1 mm isotropic margin was added to the GTV to account for setup errors, resulting in a planning target volume (PTV) of 0.878 cm³. A GRS treatment plan was developed using 12 isocenters to ensure adequate PTV coverage. A total of 286 non-coplanar beams were directed at the isocenter coordinates. A dose of 21 Gy in a single fraction was prescribed to the 54% isodose line at the PTV margin. Ocular akinesia was achieved using short-term general anesthesia. Treatment was delivered using the ZAP-X system, with a total treatment time of 59 minutes and 45 seconds. The delivered monitor units (MUs) totaled 23,339.47. The patient tolerated the procedure well, with no immediate post-treatment complications. Follow-up evaluations were conducted at six weeks and at three and a half months post-treatment. The patient reported no vision loss; however, fundus examination of the left eye at three and a half months revealed signs of macular edema. Regular ocular assessments and follow-up imaging are planned to monitor treatment response and evaluate long-term outcomes.

This case report highlights ZAP-X as a potential alternative to established SRS modalities for the treatment of juxtapapillary choroidal melanoma. It also underscores the need for larger studies to evaluate the efficacy and safety of ZAP-X in the management of choroidal melanoma.

## Introduction

Uveal melanomas are relatively rare but potentially life-threatening ocular tumors. They arise from melanocytes of neuroectodermal origin located in the stroma of the uveal tract, which includes the iris, ciliary body, and choroid. Among these, choroidal melanoma is the most frequently diagnosed (90%), followed by ciliary body melanoma (6%) and iris melanoma (4%). Although uveal melanomas account for only 5% of all primary melanomas, they are the most common primary intraocular tumors in adults, with a mean age-adjusted incidence of 5.1 cases per million per year [1]. These tumors are most commonly observed in older individuals (59-62 years) in Europe and the United States but tend to present at a younger age in Asian populations (mean age of 46 years in Asian Indians and 45 years in Chinese patients). These tumors are more prevalent in the Caucasian population, and the age-adjusted incidence is higher in males than in females. Additional risk factors associated with uveal melanoma include cutaneous nevi, cutaneous freckles, iris nevi, and ocular or oculodermal melanocytosis [2].

Anterior uveal melanomas (iris melanomas) are typically diagnosed 10-20 years earlier than posterior uveal melanomas (ciliary body and choroidal melanomas) and are associated with a better prognosis. They are often incidental findings and commonly present as changes in iris color (heterochromia) or pupillary distortion (corectopia). On the other hand, posterior uveal melanomas typically present with blurred vision (38%), photopsia (9%), floaters (7%), visual field loss (6%), visible tumor (3%), pain (2%), and metamorphopsia (2%). In 30% of cases, patients are asymptomatic [2]. Tumors located in the periphery may reach considerable size before causing visual field loss, whereas those near the macula or optic disc often lead to early visual loss due to cystic macular edema or secondary retinal detachment [1]. These tumors have a high tendency to metastasize, with an overall risk of 50%, depending on patient age, tumor size, and the presence of subretinal fluid, hemorrhage, or extraocular extension [3]. The most common sites of metastasis are the liver (89%), lungs (29%), bones (17%), subcutaneous tissue (12%), and lymph nodes (11%) [2]. Metastasis is associated with high mortality, and despite recent advances in diagnosis and treatment, uveal melanomas remain life-threatening, with an overall mortality rate of 40-50% at 15 years, primarily due to liver metastasis [4].

The management of posterior uveal melanomas (mainly choroidal melanomas) depends on several factors, including tumor location, extent, size, visual acuity at presentation, the condition of the uninvolved eye, patient age, and overall general health. Treatment options for localized disease include direct laser photocoagulation, transpupillary thermotherapy (TTT), photodynamic therapy, surgery (resection, enucleation, and orbital exenteration), and radiation therapy [2].

Juxtapapillary choroidal melanomas are described as tumors in which the posterior tumor margin is 2.5 mm or less from the optic disc, touching the optic disc, or partially or completely surrounding the disc [5]. Owing to their proximity to the macula and optic nerve, their management carries a higher risk of ocular complications, including vision loss.

Radiation therapy options for choroidal melanomas include plaque brachytherapy (using iodine-125, ruthenium-106, or palladium-103), fractionated proton beam therapy, and stereotactic photon beam radiation therapy (Gamma Knife and CyberKnife-based stereotactic radiosurgery (SRS), and linear accelerator (LINAC)-based hypofractionated stereotactic radiation therapy (SRT)) [2]. SRS is a well-established treatment modality for choroidal melanomas. It offers the advantage of single-session delivery without the need for invasive surgery or prolonged hospitalization associated with plaque brachytherapy, and it eliminates the extended treatment duration required for hypofractionated SRT.

The ZAP-X Gyroscopic Radiosurgery System offers a cutting-edge platform for the delivery of SRS in tumors of the brain, head, and neck. Its standout feature is its self-contained, self-shielded design, which eliminates the need for a shielded radiation vault. It incorporates a 3-MV S-band LINAC, which is mounted on a gyroscope-like gantry with independently rotating dual axes around a unique common isocenter. The system delivers non-coplanar beams by moving the radiation source along a virtual spherical surface, covering approximately 2π steradians of solid angle. In addition, it features a 45 cm source-to-axis distance (SAD), which helps reduce radiation leakage and sharpens the beam penumbra. It offers a dose rate of up to 1,500 MU/minute and provides circular collimation with diameters ranging from 4 mm to 25 mm. The collimator size is automatically adjusted during treatment via a novel tungsten wheel collimator. For image guidance, ZAP-X employs gantry-mounted kilovoltage imaging, which captures images from specific angles to achieve precise initial skull alignment. The system facilitates continuous image acquisition at predetermined intervals during treatment delivery. Skull offsets are calculated by aligning captured images with real-time digitally reconstructed radiographs, and the isocenter position is corrected by repositioning the patient. ZAP-X, with its lower beam energy and shorter SAD, exhibits characteristics that closely align with those of the Gamma Knife. A study focused on the peripheral dose fall-off of ZAP-X indicated that its beam characteristics are more similar to those of the Gamma Knife [6].

SRS requires effective techniques for ocular immobilization to ensure the precise delivery of radiation. These techniques include retrobulbar anesthesia, bridle sutures through the rectus muscle to maintain eye position, vacuum suction cups, and television camera systems with computer-assisted eye tracking and automated gating [7]. As an alternative, short-term general anesthesia not only provides ocular akinesia for SRS but also ensures overall patient immobilization, while alleviating anxiety and discomfort during treatment delivery.

We report a case of juxtapapillary choroidal melanoma treated using the ZAP-X Gyroscopic Radiosurgery System. This report aims to demonstrate the feasibility of the procedure and share our initial institutional experience with this treatment modality.

## Case presentation

A 46-year-old female patient with no known medical comorbidities presented with complaints of flashes of light and floaters in the left eye for two months. She denied any history of trauma, headache, vomiting, or other associated symptoms.

Clinical evaluation

**Figure 1:**
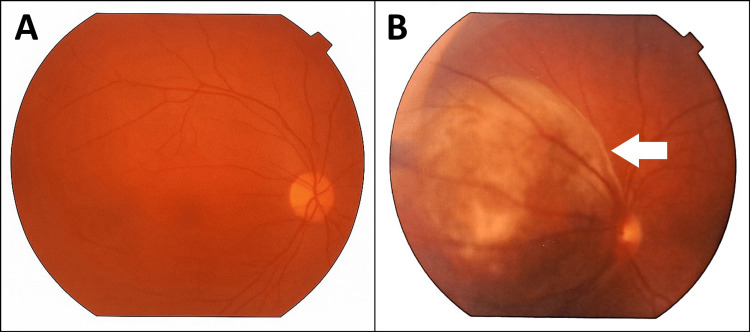
Fundus examination showing a normal right eye (A) and a large choroidal mass in the left eye (B), indicated by a white arrow.

**Figure 2:**
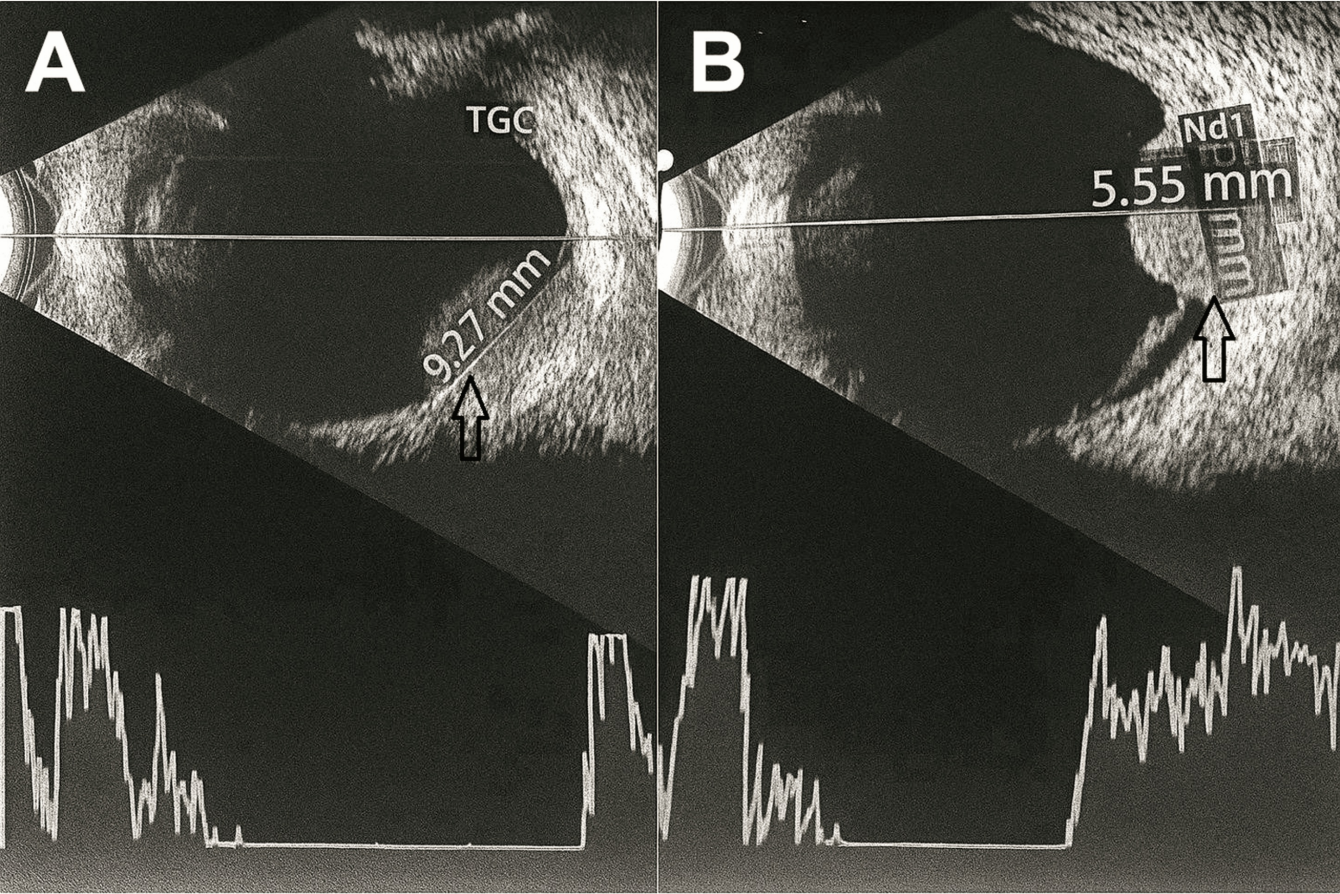
Ultrasound B-scan of the left eye demonstrating a choroidal mass with a basal diameter of 9.27 mm (A) and a thickness of 5.55 mm (B), indicated by black arrows.

**Figure 3:**
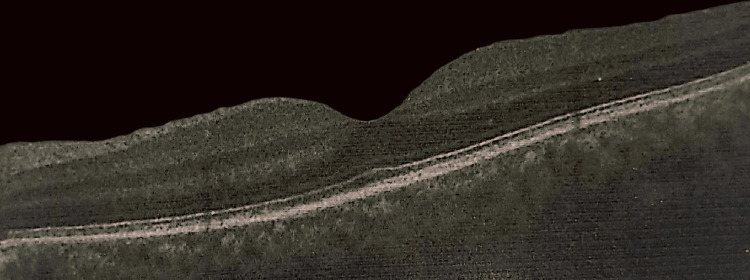
Unremarkable optical coherence tomography of the left retina.

**Figure 4:**
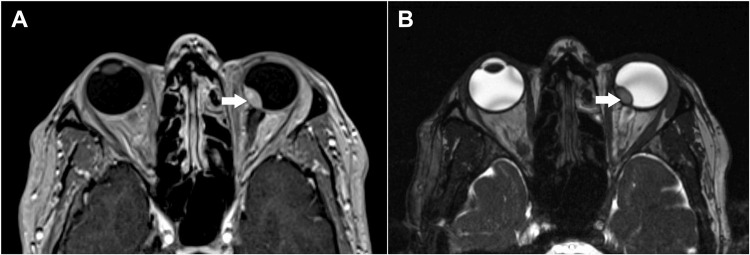
MRI of the orbit: Axial images showing a T1 hyperintense (A) and T2 hypointense (B) lesion in the posteromedial, juxtapapillary location of the left eye, indicated by white arrows.

**Figure 5:**
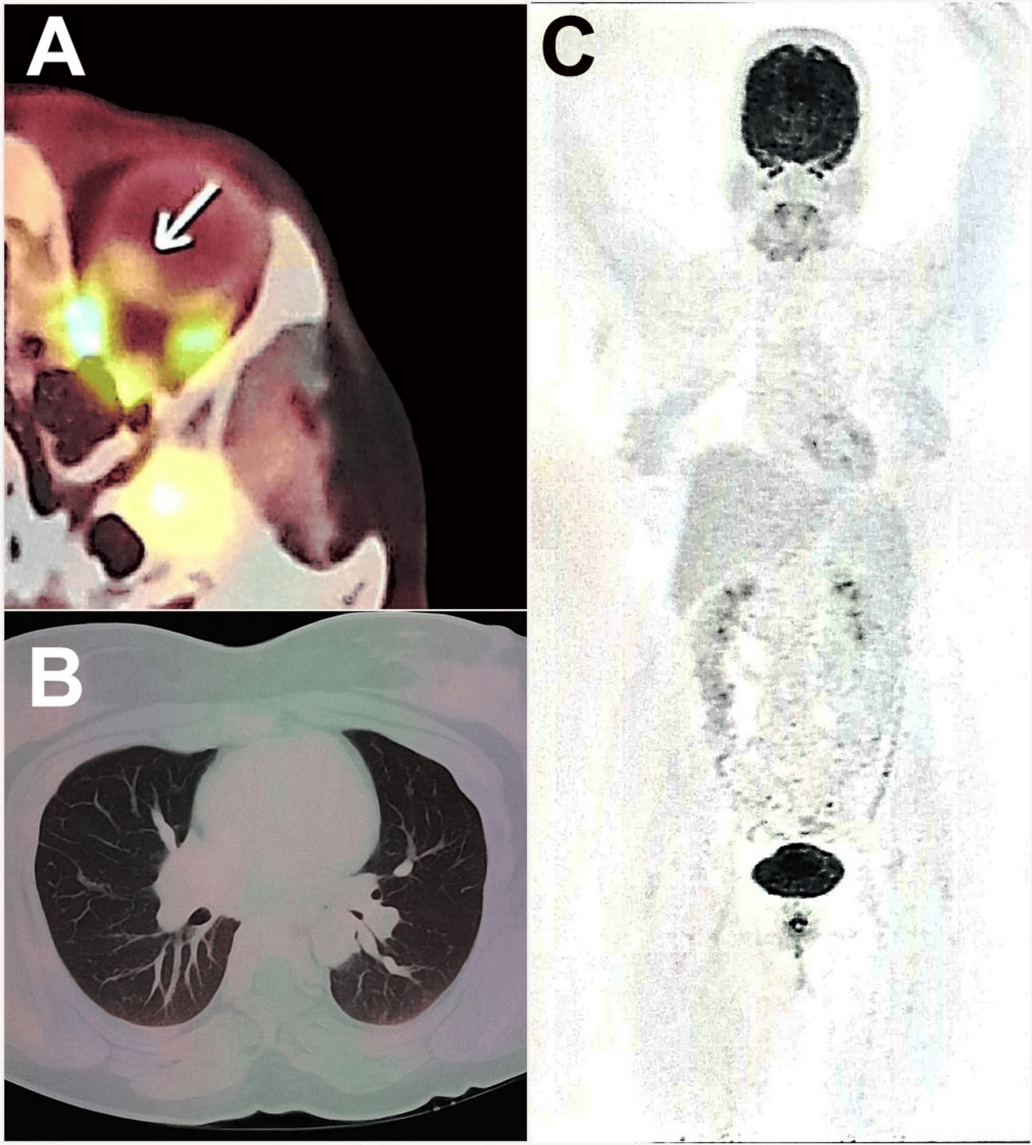
18F-fluorodeoxyglucose positron emission tomography-computed tomography scan: Mildly fluorodeoxyglucose-avid lesion in the left eye, indicated by a white arrow (A), with no other lesions detected in the surveyed regions of the body (B and C).

Pupillary examination revealed both pupils to be 2 mm in size and reactive to light. Visual acuity was 6/6 in both eyes. Anterior segment examination and intraocular pressure measurements were unremarkable in both eyes. Fundus examination of the left eye revealed a large choroidal mass nasal to the optic disc, involving the nasal half of the disc (Figure 1). The fundus of the right eye was normal. Ultrasound B-scan of the left eye showed a highly reflective, dome-shaped mass with high surface reflectivity and medium homogeneous internal reflectivity, measuring 9.27 mm in basal diameter and 5.55 mm in thickness, located nasal to the disc in the peripapillary area (Figure 2). Optical coherence tomography (OCT) of the left retina was unremarkable (Figure 3). The patient subsequently underwent contrast-enhanced MRI of the orbits, which demonstrated a broad-based, oval-shaped enhancing lesion along the posteromedial wall of the left eyeball, extending up to, but not completely covering, the optic nerve head. The lesion measured 9.0 × 8.3 × 4.4 mm in maximum transverse, superoinferior, and anteroposterior dimensions, respectively. It appeared hypointense on T2-weighted images and hyperintense on T1-weighted images. There was no evidence of extraocular extension, and the vitreous showed normal signal intensity. Both optic nerves were of normal thickness and signal intensity. The retro-orbital fat appeared unremarkable. The findings were suggestive of melanoma (Figure 4). A staging workup to rule out metastasis was subsequently performed. An 18F-fluorodeoxyglucose positron emission tomography-computed tomography (FDG PET-CT) scan demonstrated a faintly FDG-avid (maximum standardized uptake value of 2.49), enhancing, broad-based, oval-shaped lesion measuring 7 × 4 mm along the posteromedial wall of the left eyeball, extending to the origin of the optic nerve head, without extraocular extension. No other significant FDG-avid lesions were identified in the surveyed regions of the body (Figure 5).

After assessment of all relevant investigations, a diagnosis of non-metastatic, localized left eye juxtapapillary choroidal melanoma was made. The tumor was classified as medium-sized according to the Collaborative Ocular Melanoma Study (COMS) [8] and staged as T2aN0M0 per the American Joint Committee on Cancer (AJCC), 8th edition, 2017. Routine laboratory investigations, including complete blood count, liver function tests, kidney function tests, and glycated hemoglobin, were within normal limits. The disease status, prognosis, and available treatment options, along with their respective risks and benefits, were explained to the patient and her attendants. Despite treatment, the potential for disease progression, visual deterioration, and the need for enucleation of the eye, owing to the nature of the disease, was discussed. She was subsequently offered SRS using the ZAP-X Gyroscopic Radiosurgery System at our center, Indraprastha Apollo Hospital, New Delhi, India.

Treatment planning

A thermoplastic head mask (ZAP Surgical Systems Inc., San Carlos, CA, USA) was fabricated to ensure reproducible head positioning and effective immobilization. A planning CT scan was acquired using a 128-slice multidetector CT scanner (Siemens Healthineers, Erlangen, Germany) with a 1 mm axial slice thickness, with the mask in place and the patient in the treatment position. Subsequently, a planning 3 Tesla MRI scan was performed using a Biograph mMR scanner (Siemens Healthineers, Erlangen, Germany), with 1 mm 3D reconstruction. T1-weighted, T2-weighted, and gadolinium-enhanced T1-weighted sequences were acquired.

**Figure 6:**
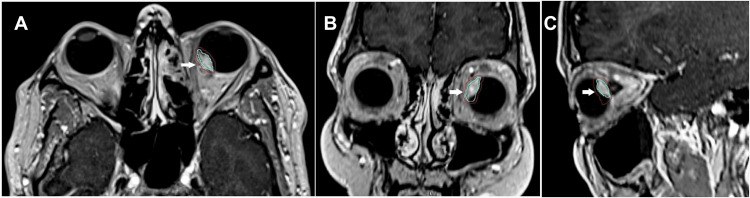
Gross tumor volume (GTV) and planning target volume (PTV) delineation on fused MRI images, indicated by white arrows, in axial (A), coronal (B), and sagittal (C) views. Blue contour represents the GTV. Red contour represents the PTV.

The CT and MRI datasets were imported into the treatment planning system (iPLAN version 4.1.6; Brainlab AG). Co-registration of the CT and MRI scans was performed, ensuring accurate fusion in the regions of interest. The gross tumor volume (GTV) was delineated on the fused CT-MRI images, resulting in a volume of 0.391 cm³. The planning target volume (PTV) was generated by applying a 1 mm isotropic margin to the GTV to account for setup uncertainties. The volume of the PTV was 0.878 cm³. Organs at risk were delineated according to standard guidelines and a consensus-based atlas [9]. The GTV and PTV volumes, as previously described, are shown in Figure 6.

**Figure 7:**
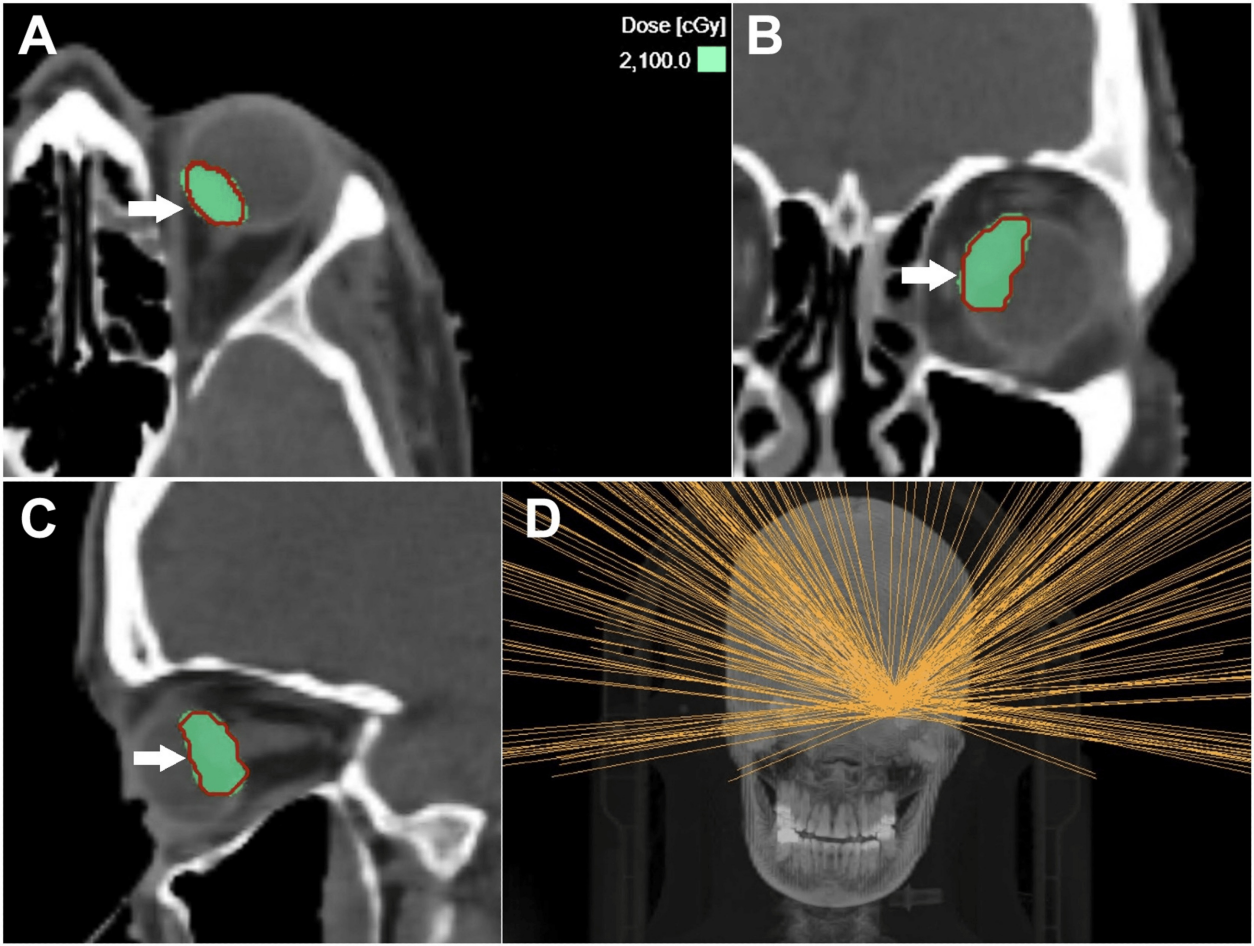
Dose wash of 21 Gy prescribed to the 54% isodose line at the planning target volume margin, shown in axial (A), coronal (B), and sagittal (C) views, as indicated by white arrows. Beam profile is depicted in (D).

**Figure 8:**
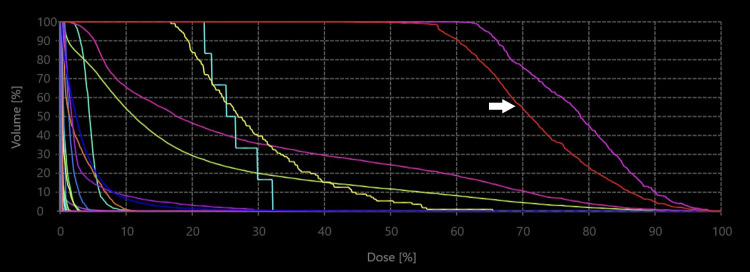
Dose–volume histogram of the treatment plan. The white arrow indicates the planning target volume.

The planning CT scan, along with the delineated structure sets, was subsequently imported into the GRS Treatment Planning System (v1.8.59; ZAP Surgical Systems Inc., San Carlos, CA, USA). The treatment plan was created using 12 isocenters to cover the PTV of 0.878 cm³: four with 4 mm, five with 5 mm, and three with 7.5 mm circular collimators. A total of 286 non-coplanar candidate beams were targeted at the isocenter coordinates. The beam weights were optimized using an inverse planning algorithm to achieve a dose distribution conformal to the PTV, with a sharp fall-off toward the surrounding normal tissues. The contralateral eye was blocked from beam traversal. A dose of 21 Gy in a single fraction was prescribed to the 54% isodose line at the margin, encircling the PTV (Figure 7). The dose-volume histogram is shown in Figure 8. The dose to the target is summarized in Table 1, and the dose to the organs at risk is presented in Table 2.

**Table 1:** Dose to the target.

Parameter	Value
Planning target volume (cm^3^)	0.878
Prescription dose (Gy)	21
Prescribed isodose (%)	54
Planning target volume coverage (%)	99.27
Maximum dose (Gy)	38.89
Minimum dose (Gy)	16.74
Mean dose (Gy)	28.12
Conformity index	1.26
Homogeneity index	1.85
Gradient index	2.82

**Table 2:** Dose to organs at risk

Organs at risk	Minimum dose (Gy)	Mean dose (Gy)	Maximum dose (Gy)
Eye_left	0.16	7.4	38.89
Lens_left	0.28	0.62	2.02
Macula_left	6.49	11.57	25.44
Fovea_left	8.5	10.27	12.49
Retina_left	0.49	11.21	38.13
Cornea_left	0.17	1.05	4.52
Lacrimal gland_left	0.72	1.77	4.23
Optic nerve_left	0.15	1.34	13.89
Optic chiasm	0.08	0.24	1.1
Brainstem	0.07	0.11	0.7
Brain	0.04	0.14	5.49

Treatment delivery

The patient provided both verbal and written informed consent after being thoroughly informed about the indication, procedure, and potential side effects of the treatment. She underwent a pre-anesthetic check-up to assess her suitability for general anesthesia. Subsequently, she underwent SRS in December 2024. Before the start of treatment, she was placed under short-term general anesthesia by the anesthesiologist, ensuring immobilization and ocular akinesia. Treatment was then delivered in a single session using the ZAP-X Radiosurgical System.

Image acquisition was performed every 45 seconds during treatment delivery. Skull offsets were monitored by aligning the captured images with real-time digitally reconstructed radiographs to ensure treatment remained within acceptable deviations. Continuous remote monitoring of the patient’s vital parameters and motion was performed throughout the session. The total treatment duration was 59 minutes and 45 seconds, resulting in the delivery of 23,339.47 MUs. The procedure was uneventful, and the patient experienced a smooth recovery from anesthesia. No immediate post-radiosurgical complications were observed. She was discharged home after 12 hours of observation following the procedure, during which she reported no visual complaints and remained clinically stable.

Follow-up

An ophthalmological assessment was performed three days after the procedure. The patient did not report any new symptoms. Visual acuity was 6/6 in both eyes. Anterior segment examination and intraocular pressure measurements were normal bilaterally. Fundus examination revealed no new abnormalities.

At the six-week follow-up, the patient continued to report occasional flashes of light and floaters, along with intermittent mild pain in the left eye. There was no change in visual acuity or other ocular findings.

**Figure 9:**
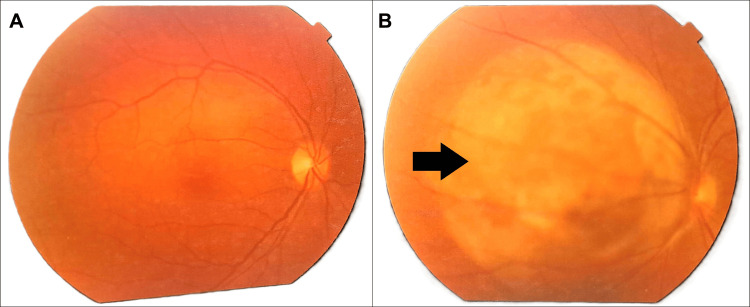
Follow-up fundus examination showing a normal right eye (A) and a persistent choroidal mass in the left eye (B), as indicated by a black arrow.

**Figure 10:**
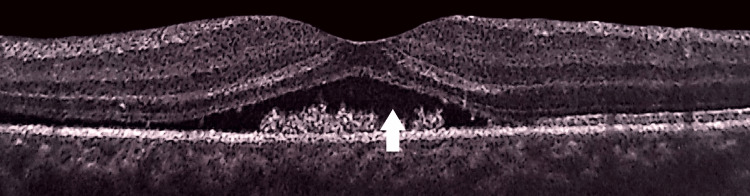
Follow-up optical coherence tomography of the left retina showing macular edema, as indicated by a white arrow.

At the three-and-a-half-month follow-up, she reported mild photophobia in the left eye, without complaints of floaters, flashes of light, or visual loss. Visual acuity was 6/6 in the right eye and 6/9 in the left eye. Anterior segment examination and intraocular pressure measurements remained normal in both eyes. Fundus examination of the left eye revealed a persistent choroidal mass nasal to the optic disc (Figure 9), along with macular edema. Notably, OCT of the left retina confirmed the presence of macular edema (Figure 10). Fundus examination and OCT findings in the right eye were unremarkable.

## Discussion

The primary objectives in the modern management of localized choroidal melanoma are to achieve globe preservation, ensure effective local tumor control, prevent recurrence and metastasis, and maximize the preservation of visual function [1]. The COMS defines choroidal melanoma based on size as follows: small (1.5-2.4 mm in apical height and 5-16 mm in diameter), medium (2.5-10 mm in apical height and ≤16 mm in diameter), and large (>10 mm in apical height and >16 mm in diameter) [8]. Before the randomized, multicenter COMS trial, the standard of care involved radical surgery, including enucleation and exenteration, with the aim of prolonging survival. However, the COMS trial demonstrated that iodine-125 (I-125) brachytherapy offers a comparable survival rate for medium-sized uveal melanomas when compared to enucleation [8]. As a result, globe preservation became both feasible and desirable. Enucleation is currently considered for patients with large tumors, poor visual potential, moderate extraocular extension, or neovascular glaucoma. Orbital exenteration is preferred in eyes with extensive extraocular extension or confirmed orbital involvement [2].

Definitive radiation therapy has become a well-established option for the treatment of choroidal melanoma in patients for whom globe preservation is feasible. I-125 plaque brachytherapy is a safe and effective treatment for small- to medium-sized choroidal melanomas [3]. Proton beam therapy and stereotactic photon beam radiation therapy are comparable to plaque brachytherapy in terms of tumor control, visual outcomes, and survival [2]. Table 3 provides a comparison of radiation treatment techniques and relevant considerations for the management of choroidal melanoma.

**Table 3:** Comparison of radiation techniques and treatment considerations in choroidal melanoma. SRS = stereotactic radiosurgery; SRT = stereotactic radiation therapy; LINAC = linear accelerator

Treatment modality	Five-year local control	Five-year survival	Treatment considerations
Radioactive seed plaque brachytherapy	90–95% [10,11]	89% [10]	Useful for small- to medium-sized tumors [3]; contraindicated or generally not considered for tumors <2 mm from the optic disc [4,11]; invasive; requires surgery, technical expertise, and hospitalization
Proton beam therapy (fractionated treatment)	97% [12]	60–87% [10]	Suitable for medium and large tumors or tumors close to the optic disc [11,13]; entails the placement of tantalum clips for eye positioning [1]; limited availability and high cost; fractionated treatment results in prolonged treatment duration
Gamma Knife (single-fraction SRS)	94–97% [10]	76% [10]	Established modality for SRS; suitable for medium and large tumors or tumors close to the optic disc; requires an invasive rigid frame for target fixation; prolonged treatment time (2–4 hours) requiring eye immobilization using invasive retrobulbar anesthesia [3,4,10,11]
CyberKnife (single-fraction SRS or hypofractionated SRT)	77–84% [14]	78.4% [13]	Suitable for medium- and large-sized tumors [13]; hypofractionated SRT is feasible for tumors close to the optic disc [15]
LINAC-based hypofractionated SRT	95.90% [12]	84.60–90.20% [12]	Suitable for large tumors and tumors close to the macula [12]; non-invasive and frameless; shorter duration of treatment per session (30 minutes versus 2–4 hours with Gamma Knife) [11]

Juxtapapillary choroidal melanoma, in particular, is difficult to treat with radiation therapy due to its proximity to critical structures involved in vision preservation, such as the optic nerve, optic disc, and macula. As a result, its treatment is associated with an increased risk of radiation-related ocular complications. Moreover, irradiation of tumors close to the optic nerve is generally associated with substantial visual loss, regardless of the type of radiation technique used [5]. All the radiation modalities listed in Table 3, except for plaque brachytherapy, have been considered feasible for treating juxtapapillary tumors, with variable treatment-related outcomes [4,10-12,15]. Each technique has its advantages and limitations. However, they all share a common goal: to achieve an acceptable balance between delivering an adequate radiation dose for tumor control and minimizing exposure to normal ocular structures.

The decision to treat juxtapapillary choroidal melanoma with a particular radiation modality, therefore, requires careful consideration of multiple factors, including institutional capabilities and patient preferences. Plaque brachytherapy, while generally considered unsuitable for juxtapapillary tumors, has been adapted for use in such cases through the application of notched plaques. It has also been combined with TTT to reduce the risk of treatment failure at the optic nerve margin [15]. However, it is invasive and may necessitate extended hospitalization. Proton beam therapy is limited by its availability, high cost, and the extended treatment duration required for fractionated delivery. SRS, on the other hand, is feasible for juxtapapillary tumors, as it allows for a sharp dose fall-off outside the treatment volume, thereby limiting radiation exposure to surrounding critical structures. It offers the advantage of delivering a high radiation dose in a single fraction, thereby completing treatment in a shorter duration compared to hypofractionated SRT. This is particularly significant, considering that ocular melanoma cell lines are reported to be relatively radioresistant at lower doses and more responsive to higher single-dose radiation delivered by SRS [11]. SRS for juxtapapillary choroidal melanoma has most commonly been performed using Gamma Knife radiosurgery [4,10]. The use of CyberKnife for SRS in this tumor group has been reported infrequently in the literature [13,15].

The ZAP-X Gyroscopic Radiosurgery System is a newer modality designed for cranial SRS. Consequently, comparative studies between ZAP-X and established platforms such as Gamma Knife and CyberKnife remain limited. Table 4 summarizes key findings from two comparative dosimetric studies, which suggest that ZAP-X may be a feasible alternative for SRS. Notably, the lower gradient index reported for ZAP-X is particularly significant when treating tumors in critical locations, such as juxtapapillary choroidal melanoma.

**Table 4:** Comparison of ZAP-X, Gamma Knife, and CyberKnife for SRS in brain metastases. SRS = stereotactic radiosurgery; CI = conformity index; HI = heterogeneity index; GI = gradient index

Study	Findings	Inference
Niu et al. [6]	CyberKnife achieved better CI and HI. ZAP-X showed higher dose heterogeneity within the target, better GI, and a shorter treatment time	Both CyberKnife and ZAP-X exhibit comparable plan quality and delivery efficiency
Wang et al. [16]	The CI of Gamma Knife is lower than that of ZAP-X and CyberKnife. ZAP-X achieved a lower GI overall, shorter treatment time than CyberKnife, and better protection of brain tissue. Lower GI indicates sharper dose fall-off outside the target volume	All three treatment modalities meet clinical treatment requirements

There is a scarcity of studies evaluating the use of the ZAP-X platform for SRS in choroidal melanoma. Foerster et al. [17] published the first case report in May 2024, describing their clinical experience and workflow in treating a case of uveal melanoma using GRS on an outpatient basis. In March 2025, Kinzl et al. published a case report detailing the treatment and one-year follow-up of a patient with uveal melanoma treated with the ZAP-X system. The authors concluded that ZAP-X can be safely utilized for SRS in choroidal melanoma and holds promise as an alternative to existing treatment modalities [18].

After careful consideration of all feasible treatment modalities in a multidisciplinary setting, we offered SRS with the ZAP-X Gyroscopic Radiosurgery System to our patient with a medium-sized juxtapapillary choroidal melanoma. During treatment planning, we applied an isotropic margin of 1 mm from the GTV to the PTV to account for setup uncertainties. A prescription dose of 21 Gy was delivered in a single fraction to the 54% isodose line at the PTV margin, consistent with the approach described by Foerster et al. [17]. This dose selection was based on the findings of Leigl et al. [14], who reported local control rates of 92% at three years and 84.3% at five years for doses of 21-22 Gy, compared to 86-89% and 77.7%, respectively, for doses of 20 Gy or less. In our study, the minimum, maximum, and mean doses within the tumor volume were 16.74 Gy, 38.89 Gy, and 28.12 Gy, respectively. This considerable dose heterogeneity, resulting in a higher mean dose, may, in principle, influence outcomes in radioresistant melanoma cell lines. The PTV coverage, homogeneity index, and gradient index were comparable to those reported previously [17].

SRS requires adequate immobilization to ensure precise treatment delivery. In our case, we used a non-invasive thermoplastic head mask, which provided effective immobilization and acceptable reproducibility of head positioning. Retrobulbar anesthesia and manual vacuum fixation have been employed for ocular stabilization in previous studies [17,18]. However, retrobulbar anesthesia is invasive, requires technical expertise, and carries a risk of ocular injury. While mechanical vacuum fixation is non-invasive, it involves a complex setup. We explored the use of general anesthesia to achieve ocular akinesia, an approach routinely used in complex ocular surgeries [19]. The short-term general anesthesia administered in our case enabled uncomplicated treatment, with smooth induction and rapid recovery. It provided adequate ocular akinesia and immobilization, while also alleviating the patient’s anxiety and discomfort during treatment delivery.

The tumor in our study partially involved the optic disc and was located in close proximity to the macula. As a result, the optic nerve, macula, and fovea received relatively higher radiation doses. This elevated dose carries an inherent risk of radiation-induced maculopathy and optic neuropathy at a later stage, potentially leading to vision loss. Two previously published studies on stereotactic radiotherapy for the management of juxtapapillary choroidal melanoma have reported a higher incidence of ocular complications, which also tend to manifest earlier following treatment [5,20].

At the three-and-a-half-month follow-up, the patient reported no loss of vision. However, fundus examination of the left eye and OCT of the left eye retina revealed evidence of macular edema, highlighting the risks associated with the treatment of juxtapapillary choroidal melanoma. Accordingly, the patient has been advised to undergo regular ocular assessments to monitor for potential treatment-related complications and evaluate visual function. Follow-up imaging has also been planned to assess treatment response, including an 18F-FDG whole-body PET-CT scan to detect any possible metastasis.

## Conclusions

SRS is a technically feasible modality for treating juxtapapillary choroidal melanomas, which pose unique challenges due to their proximity to critical ocular structures and the heightened risk of radiation-induced complications. The ZAP-X Gyroscopic Radiosurgery System offers a novel, non-invasive platform for delivering single-session SRS with dosimetric characteristics comparable to those of established systems such as Gamma Knife and CyberKnife. In our case, treatment with ZAP-X was successfully administered for a medium-sized juxtapapillary choroidal melanoma, with early follow-up demonstrating preserved vision, although early signs of macular edema were noted. While our findings support the feasibility of ZAP-X for this indication, the study is limited by its short follow-up duration. Given the known risks of long-term ocular toxicity associated with the treatment of juxtapapillary tumors, extended follow-up is essential to evaluate both safety and efficacy. Larger, multicenter studies are warranted to further define the role of ZAP-X in the management of choroidal melanoma, particularly for tumors located in anatomically challenging regions.
